# Pernicious Anemia Presented with Isolated Nominal Dysphasia in Type Ill Polyglandular Failure Female Patient

**DOI:** 10.7759/cureus.3306

**Published:** 2018-09-14

**Authors:** Adela H Elamami, Nafesa Elmehdwi, Eman Z Younis, Hamid Zwawi, Rabha Elsahli, Omar B Latiwesh, Azhar Hussain

**Affiliations:** 1 Internal Medicine, University of Benghazi, Benghazi, LBY; 2 Laboratory Medicine, University of Benghazi, Benghazi, LBY; 3 Medical Laboratory, Higher Institute of Medical Professions, Benghazi, LBY; 4 Medicine, Xavier University School of Medicine, Oranjestad, ABW

**Keywords:** polyglandular failure type lll, pernicious anemia, nominal dysphasia, vitamin b12 deficiency

## Abstract

Pernicious anemia (also known as Biermer’s disease) is an autoimmune atrophic gastritis which predominantly affects the fundus of the stomach. It results in a deficiency of vitamin B12 (cobalamin) as it affects the normal process of absorption in the ileum. The pernicious anemia is characterized by a wide range of hematological and neurological features. Neurological features can present without hematological manifestations. One of the early neurological features of this anemia is nominal dysphasia (word-finding difficulties), which was usually not reported before as an isolated finding. We present a case of pernicious anemia with isolated nominal dysphasia responding dramatically to parenteral vitamin B12 therapy.

## Introduction

Polyglandular autoimmune (PGA) syndromes are rare polyendocrinopathies, characterized by destruction of endocrine glands as well as other organ systems. It is caused by an immune-mediated attack on endocrine tissues [1]. Other associated disorders may include, myasthenia gravis (MG), celiac disease, and pulmonary hypertension [2,3].

The PGA is divided into four different types. The PGA type I is a monogenic autoimmune syndrome, which is caused by a defect in the AIRE (autoimmune regulator) gene located on chromosome 21. The symptoms often begin in childhood and classically characterized by a triad of chronic mucocutaneous candidiasis, hypoparathyroidism and Addison's disease [4,5]. The PGA type I is an autosomal recessive disorder also known as APECED (autoimmune polyendocrinopathy, candidiasis, and ectodermal dystrophy) or MEDAC (multiple endocrine deficiency autoimmune candidiasis syndrome) [1].

The PGA type II usually occurs in adults and is characterized by the adrenal insufficiency with autoimmune thyroid disease (Schmidt’s syndrome) and/or type I diabetes (Carpenter’s syndrome) [1,5]. The PGA type III is composed of autoimmune thyroid diseases associated with other autoimmune conditions, such as immunogastritis, pernicious anemia, type IA diabetes mellitus, vitiligo, and alopecia with the exception of Addison’s disease [1]. The PGA type IV includes all other endocrine disorders that are not included in the previous groups [6].

Pernicious anemia (PA) is an organ-specific autoimmune disorder. It is characterized by atrophic gastritis, predominantly the fundus and body of the stomach, resulting in loss of parietal cells from the gastric mucosa, submucosal lymphatic infiltrate, as well as circulating gastric parietal cell autoantibodies [1,7]. Pernicious anemia is one of the most common causes of vitamin B12 deficiency due to its malabsorption. The absorption of vitamin B12 requires a sequence of orderly events before it can be absorbed in the ileum. The vitamin B12 is released from the food source in the stomach where it binds to the haptocorrin, commonly known as R-protein. This protein is secreted by the salivary glands in the oropharynx as well as the gastric mucosal cells within the stomach. The parietal cells secrete hydrochloric acid (HCl) as well as intrinsic factor. The pancreatic proteases degrade the R-protein, freeing the vitamin B12 so that it can bind to intrinsic factor. This factor is a transport protein important for taking vitamin B12 to the ileum where it may be absorbed [8,9]. The lack of intrinsic factor, as seen in pernicious anemia, impairs the absorption of vitamin B12. Anti-intrinsic factor antibodies are a useful diagnostic tool for PA [8,10]. The main hematological manifestation is macrocytic, megaloblastic anemia. Other hematological manifestations have also been commonly reported such as neutropenia, thrombocytopenia, pancytopenia, intramedullary hemolysis due to ineffective erythropoiesis [11]. The PA also can present with diverse neurological abnormalities without hematological features in 30% of cases [12-14]. These neurological features include reversible dementia, subacute combined degeneration of the spinal cord, nominal dysphasia and dorsal column dysfunction [9].

The anomic aphasia, also known as nominal dysphasia, or amnesic aphasia, is a mild, fluent type of aphasia where the individual has difficulty retrieving the right words and failure to express themselves [15]. However, they may often be able to describe an object in detail or demonstrate how the object is used but cannot name the object [16]. Frequently used words are somewhat easier to retrieve as compared to less frequently used words [17].

## Case presentation

A 38-year-old female, single, Libyan, graduated from High Institute, had a history of type I diabetes mellitus and primary hypothyroidism for past 12 years and epileptic episodes for seven years. She is currently taking thyroxine 150 µg, basal-bolus insulin analog regimen, and Keppra (Levetiracetam) 500 mg once daily with good follow-up to neurology and endocrine clinic with reasonable control. The patient had a long history of fatigue and dizziness with documented low blood pressure for which she was screened for Addison’s disease twice. The results were negative for both tests. On 3rd August 2015, she complained of forgetting names of objects despite retaining ability to recognize the function of the objects (such as the name of mobile). We had a high degree of suspicion for nominal dysphasia. All other neurological assessments were normal including peripheral sensation testing and other memory features. The magnetic resonance imaging (MRI) of the brain, vitamin B12 levels, and complete blood count (CBC) with peripheral blood film tests were ordered. The results showed normal brain MRI; however, the levels of vitamin B12 were 122.8 pg/ml which was significantly low. To confirm the low levels of vitamin B12, the test was repeated. The vitamin B12 results were 97 pg/ml which was lower than the first test. The CBC was nearly normal as well as normal mean corpuscular volume (MCV). The peripheral blood film showed no evidence of megaloblastic changes or hypersegmented neutrophils. The lab findings, which were sent outside Libya, showed the presence of gastric parietal cell antibodies. The upper gastrointestinal (GI) endoscopy was normal. Table 1 shows the baseline analysis as well as the results of the patient serum profile. The parenteral vitamin B12 therapy was started, and the patient showed improvement in name retrieval for objects after two weeks into the treatment. On three months follow-up after the initiation of treatment, the patient recovered completely and her vitamin B12 levels were within the upper reference range.

**Table 1:** Serum baseline investigation of the patient and normal values.

Baseline Investigations	Normal Lab Values	Results
White Blood Count (WBC)	4.5–11.0 x 10^9^/L	7.4 × 10^9^/L
Hemoglobin (Hb)	Male: 13.5–17.5 g/dL, Female: 12.0–16.0 g/dL	12.5 g/dL
Mean Corpuscular Volume (MCV)	80–100 fL	84.7 fL
Mean Corpuscular Hemoglobin (MCH)	25.4–34.6 pg/cell	30 pg/cell
Mean Corpuscular Hemoglobin Concentration (MCHC)	33–36 g/dL	35.5 g/dL
Platelet Count	150–400 x 10^9^/L	201 × 10^9^/L
Vitamin B12 level	197–866 pg/ml	First test: 122.8 pg/ml, Second test: 97 pg/ml
Gastric Parietal Cell Antibodies	Normal < 40	40 (positive)
Erythrocyte Sedimentation Rate	Male: 0–15 mm/h, Female: 0–20 mm/h	20 mm/h
Thyroid Stimulation Hormone	0.5–5.0 µU/mL	2.5 µU/mL

## Discussion

The neurologic features of vitamin B12 deficiency are attributable to pathology in the peripheral and optic nerves, posterior and lateral columns of the spinal cord (subacute combined degeneration), and in the brain. An inverse correlation in the severity of hematologic and neurologic manifestations was noted. Around 27.4% lack anemia or macrocytosis [16,18].

There is a lag period of 10 years to diagnose pernicious anemia in about 14% of patients and one-third of patients were symptomatic for one year before the diagnoses were made [6,19]. Most of the individuals (99%) reported a range of general symptoms, predominantly tiredness (96%) but also, waking up tired (87%), dry skin (58%), brittle nails with (47%) or without ridging (37%), flushes or fever (43%), glossitis (34%), hair loss or greying (30%), weight loss (21%) and jaundice (6%). The remaining 1% reported no symptoms and their vitamin B12 deficiency may have been an incidental finding.

Most individuals (98%) also reported a range of neurological symptoms including memory loss (78%), poor concentration (75%), clumsiness (66%), pins and needles (66%), poor sleep (64%), confusion (62%), dizziness (59%), headaches (52%), nominal aphasia (word-finding difficulties) (50%), shoulder bumps (frequently bumping into objects as a result of balance problems) (48%), unable to stand with eyes closed (Romberg’s test) (34%), Grierson syndrome (33%) and vertigo (33%). Cardio-respiratory symptoms were reported by 86% of individuals, comprising shortness of breath (73%) and palpitations (56%) [20]. Isolated cases of nominal aphasia were reported in patients with temporoparietal tumor and stroke [6,8].

The nominal dysphasia usually occurs with other symptoms either neurological or hematological in patients with vitamin B12 deficiency. To our knowledge, the presentation of vitamin B12 deficiency as an isolated nominal dysphasia was never reported before. This was considered to be a unique finding in our patient. The rapid response of dysphasia to parenteral vitamin B12 therapy supports the concept of Hooper study (2014) which showed the dysphasia was common when early symptoms of vitamin B12 deficiency are presented [18,20].

The long history of fatigue, dizziness and low blood pressure in our patient may be explained by vitamin B12 deficiency. This was confirmed with a negative screening test for Addison’s disease and the patient’s symptoms improved after vitamin B12 replacement.

In dominant temporal lobe epilepsy, patients typically show consistent and quasi-systematic impairment of both object and famous people naming when compared to controls. Despite our patient’s seven years history of epilepsy, the patient was well-controlled with no episodes for the past four years, making epilepsy a cause of the dysphasia highly unlikely [8,19].

## Conclusions

The direct link to nominal dysphasia in our patient was found to be due to vitamin B12 deficiency. This was confirmed when symptoms of nominal dysphasia were normalized with B12 replacement. Prior to this case report, an isolated case of nominal dysphasia, primarily affected by a vitamin B12 deficiency, was a rare occurrence. Our case study provides a strong correlation to these two pathologies. We conclude that the long-term deficiency of vitamin B12 causes neurological damage, although the exact mechanism by which it does this is not fully understood.
